# A DNA Polymerase Subunit Gamma (POLG) Mutation Imposing a Difficult Differential Diagnosis of Hepatic Encephalopathy in a Newborn: A Case Report

**DOI:** 10.7759/cureus.65239

**Published:** 2024-07-24

**Authors:** Ithamar Cheyne, Bartosz Boryszewski, Wyven Chang, Małgorzata Mikaszewska- Sokolewicz

**Affiliations:** 1 Anesthesiology and Critical Care Scientific Circle English Division (ANKONA ED), Medical University of Warsaw, Warsaw, POL; 2 Anesthesiology and Intensive Care, Children's Memorial Health Institute, Warsaw, POL

**Keywords:** polg, differential diagnosis, genetic predisposition to disease, case report, hepatic encephalopathy

## Abstract

Hepatic encephalopathy (HE) is a condition connected with neuropsychiatric alteration during hepatic failure. The differential diagnosis of HE is challenging due to overlapping symptoms with other conditions. Polymerase subunit gamma (POLG) is a mitochondrial gene, and an infant POLG mutation can manifest with severe and progressive hepatic failure and encephalopathy, imposing a difficult differential diagnosis due to similarities to other conditions. The lack of curative treatment leads to a poor prognosis.

An 11-month-old boy was admitted to the intensive care unit (ICU) due to altered consciousness and increasing edema due to acute hepatic failure of unknown etiology. After extensive multidisciplinary discussions and a lack of response to treatment for more than three weeks, a mitochondrial disease was suspected, and a genetic test was taken. The patient’s condition continued to deteriorate. The patient died on the 25th day of hospitalization in the ICU. After death, a genetic test confirmed a rare POLG mutation NM_002693.3(POLG):c.3104+2T>A (Variation ID: 422378 Accession: VCV000422378.8).

We suggest that a screen test for POLG mutations be considered early in the diagnostic process and that clinicians consider mitochondrial genetic mutations, such as POLG mutations, more often. This article is the first to describe a patient with this specific mutation.

## Introduction

Hepatic encephalopathy (HE) is a neuropsychiatric syndrome associated with hepatic failure and shunting of intestinal blood flow [1]. There are few pathomechanisms involved in HE. The main one is decreased liver clearance of ammonia and other intestinal neurotoxins, which can cause an increase in neuro-inhibition by agonism of GABAergic transmission [2]. Other possible mechanisms include increased gamma-aminobutyric acid (GABA) penetration through the blood-brain barrier, resulting in inhibitory GABAergic transmission and manganese intoxication [1]. The diagnosis of HE is primarily clinical.

Human pol γ is composed of POLG, one of the nuclear genes associated with mtDNA depletion or deletion disorders. Mutations in polymerase subunit gamma (POLG) are the most common single-gene cause of mitochondrial disease in general and are the most frequent cause of mitochondrial epilepsy at all ages [3]. The most documented POLG-related disorders during neonatal and infantile periods are the myocerebrohepatopathy spectrum (MCHS) and Alpers-Huttenlocher syndrome (AHS). Treatment of those syndromes is symptomatic and palliative [4]. 

This case report explores the differential diagnosis of HE and emphasizes the importance of including POLG mutation testing. 

## Case presentation

An 11-month-old male (XY) patient was admitted to the intensive care unit (ICU) due to altered consciousness and increasing edema during acute hepatitis of unknown origin. The patient is a firstborn child of homogamous parents of European (non-Finnish) descent. He was delivered via cesarean section at 37 weeks of gestation due to maternal hemolysis, elevated liver enzymes, and low platelets (HELLP) syndrome, with a birth weight of 2580 grams and an appearance, pulse, grimace, activity, and respiration (APGAR) score of 10. Up until nine months of age, the patient demonstrated typical developmental progress.

At nine months old, the patient was admitted to a lower reference center and was diagnosed with encephalitis of undetermined etiology, manifested as symptomatic epilepsy with sporadically elevated transaminase levels, which, at the time, were linked to the antiepileptic treatment. The patient was said to be in good overall health and was discharged to rehabilitation after a few days.

At eleven months of age, the patient was admitted again to a lower reference center due to severe emesis with brown content. Imaging studies demonstrated distension of intestinal loops on abdominal X-ray, and laboratory investigations unveiled elevated inflammatory markers, transaminases (ALT 121 IU/L, AST 318 IU/L), total bilirubin (3.9 mg/dL), direct bilirubin (1.8 mg/dL), prolonged international normalized ratio (INR) (3.1), and activated partial thromboplastin time (aPTT) (46 seconds). Therapeutic measures included the administration of antibiotics (ceftriaxone, vancomycin, and metronidazole), cryoprecipitate, and IV acyclovir. The patient was subsequently transferred to the ICU of the lower reference center two days later due to suspicion of sepsis from an undetermined source. Ultrasound was performed two days after admission to the ICU, revealing intussusception on the intestine, which was addressed surgically.

Escalating indicators of liver failure, evidenced by prolonged INR and increased bilirubin levels, led to the transfer of the patient to the gastroenterology clinic in our hospital. In the gastroenterology department, the patient developed ascites and worsening pleural effusions that compromised breathing. In addition, glycemic swings required corrections, and decreasing diuresis called for more intensive treatment. A cooperative, multi-specialist approach was used to assess the likelihood of a metabolic disease and liver transplant candidacy. The decision to admit the patient to the ICU was taken to provide thorough management, including continuous renal replacement therapy.

**Figure 1:**
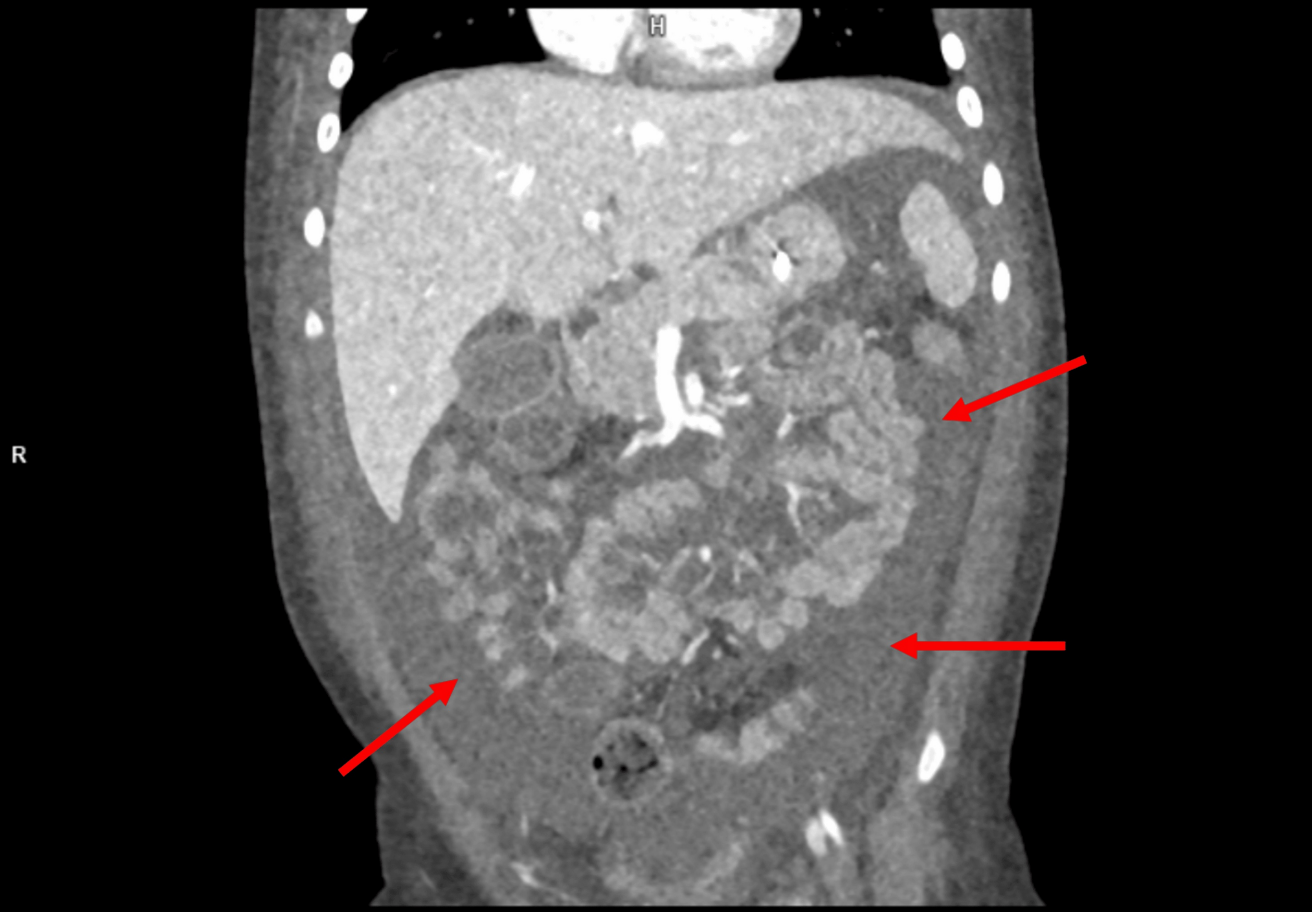
Abdominal CT scan showing diffuse free fluid in the abdominal cavity. The red arrows indicate free abdominal fluid.

**Figure 2:**
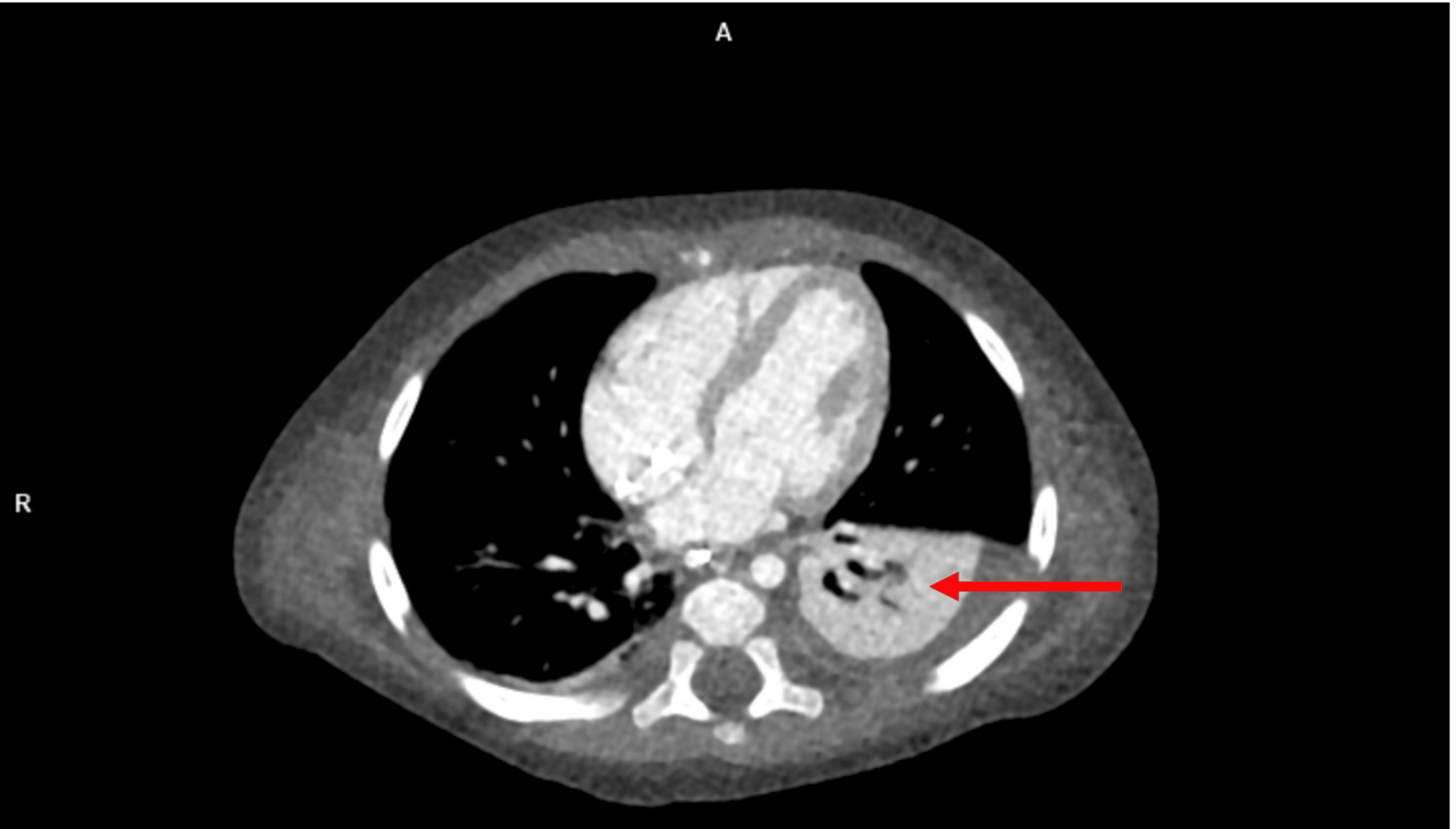
Non-contrast lung CT scan with a red arrow showing free fluid accumulation.

After the patient was transferred to the ICU, a physical examination revealed a Glasgow Coma Scale (GCS) of 6-7, showing symmetrical pupils with poor reactions to light and reaction only to painful stimuli. Further examination revealed crackles in both lungs, hypothermia, and an abdomen arched over the chest with visible ascites in addition to inaudible peristalsis. Laboratory diagnostics showed anemia, elevated liver transaminases, lactate and ammonia, reduced serum creatinine, and hypocalcemia. Increased concentrations of alanine (488 umol/L) and proline (258 umol/L), as well as amino acids secondary to liver damage (tyrosine and methionine), were noted. On abdominal ultrasound, hepatomegaly, edema, and ascites were observed, and a trans-fontanel ultrasound showed widened paracerebral space. Echocardiography did not reveal a pathological amount of fluid in the pericardium, and contractility was preserved. To exclude tumors, a head and torso CT scan was performed, revealing swelling of tissues and fluid in the pleural and abdominal cavities but no visible masses (Figures 1, 2). Initial treatment in the ICU included antibacterial and antiviral therapy, and treatment for edema with dexamethasone and mannitol was continued. Red blood cell transfusions were administered to address anemia, and active warming measures were implemented to address hypothermia. The patient was placed in a medically induced coma and received treatment to manage pain. Continuous veno-venous hemodialysis (CVVHD) renal replacement therapy was initiated upon admission to the ICU. Throughout the hospital stay, the patient underwent therapy involving different diuretics and correcting ion imbalances. Due to low serum carnitine levels, the patient was also supplemented with IV carnitine (50 - 100 mg/kg/day) in three divided doses. Plasma preparations and antithrombin III were administered to manage coagulation irregularities, along with parenteral feeding provided to the patient.

**Figure 3:**
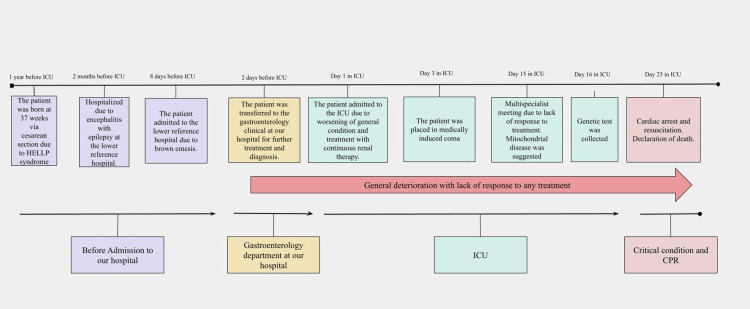
Timeline of the major events in a patient with hepatic encephalopathy due to rare POLG mutation HELLP: hemolysis, elevated liver enzymes, and low platelets syndrome; ICU: intensive care unit; CPR: cardio-pulmonary resuscitation

 Due to the patient's severe condition (Figure 3), a broad differential diagnosis approach was taken.

Metabolic differential diagnosis was considered, and several laboratory tests were performed. Tests for congenital glycosylation disorders (CGD) were performed, including Tandem mass spectrometry (MS/MS) and acylcarnitine profile, which showed reduced C0 (6.55 µmol/L) and slightly increased C4 and C14-OH indicative of liver damage. Gas chromatography/mass spectrometry (GC/MS) of urine revealed elevated C6-10 3-hydroxydicarboxyl aciduria with increased ketonuria, metabolites of drugs of the prescribed medications, and elevated p-hydroxyphenyl acetic acid and p-hydroxyphenyl pyruvic acid- not indicative of metabolic disorders. Upon oncological consultation, hemophagocytic lymphohistiocytosis syndrome was considered. However, a bone marrow biopsy revealed no signs of any oncological diseases. Head and torso CT did not show any findings aligning with cancer. 

A consultation with a nephrologist was sought concerning the escalating edema, prompting a kidney ultrasound. Although nephrological etiology was initially considered, it appeared improbable upon assessment.

Multiple probable infectious pathogens were screened for, such as hepatitis A virus (HAV), hepatitis B virus (HBV), hepatitis C virus (HCV), cytomegalovirus (CMV), serum bacteria, and fungi. Notable findings were elevated Rubella IgG (27,80 UI-pos > 10), with no active Rubella infection. Herpes simplex virus (HSV)-1 IgG was slightly elevated (12,80 NTU-pos > 11), and polymerase chain reaction (PCR) confirmed no active HSV-1 infection. Epstein-Barr virus (EBV) IgG was elevated (26.46 S/CO- pos < 0.75), but the PCR test returned negative for active infection. The Toxoplasma gondii IgG was slightly elevated, but the IgM test was negative.

Alternative potential diagnoses included hematological and autoimmune etiologies, which were subsequently excluded from consideration.

The condition of the patient continued to deteriorate. Increase in generalized edema, jaundice, and head circumference, in addition to worsening liver functions and other lab tests, resulting in a liver transplant qualification process. The patient experienced a myoclonic seizure that was initially addressed with midazolam, which proved ineffective, prompting the administration of clonazepam (0.5 mg IV), which successfully halted the seizure. A clonidine infusion was subsequently initiated, and modifications to the antiepileptic regimen eventually consisted of phenobarbital, levetiracetam, and vitamin B1. After the myoclonic seizure, the patient was disqualified for a liver transplant. Due to the severe condition and lack of therapeutic response, a multidisciplinary meeting occurred. In the meeting, the possibility of mitochondrial disease was probable, and a fast-track new-generation sequencing (NGS) genetic test was recommended, in addition to a liver biopsy after obtaining the result of the genetic test. 

Despite extensive therapeutic efforts and a comprehensive differential diagnosis approach, no definite diagnosis was found, and the patient continued to deteriorate. On the 25th day of ICU hospitalization, the patient's heart rhythm changed from bradycardia to asystole. Despite exhaustive resuscitation efforts by the medical team, the patient was pronounced dead at 3:40 p.m. The genetic test result was received post-mortem and showed a single nucleotide variant NM_002693.3(POLG):c.3104+2T>A (Variation ID: 422378 Accession: VCV000422378.8), indicative of a POLG mutation. The POLG mutation was confirmed post-mortem, limiting the ability to assess the impact of an earlier diagnosis on treatment and prognosis.

## Discussion

The patient presented with acute hepatic encephalopathy of unknown etiology. The etiologies of such presentations are vast. The POLG mutation is one of the least commonly seen etiologies [5]. Gestational alloimmune liver disease (GALD), infection, metabolic disorders, and oncological etiology were all excluded throughout the diagnosis while the patient continued to deteriorate despite appropriate treatment. Finally, a genetic test was ordered, and a POLG genetic mutation was found. Our diagnostic approach, prioritizing broad diagnostic assessment while performing tissue biopsy and genetic testing, is in accordance with multiple studies [6,7]. An important consideration is the potential interaction of valproic acid for treating seizures during hepatic encephalopathy. Valproic acid exacerbates liver failure in the presence of a POLG mutation, and one should refrain from using it unless a POLG mutation is excluded [8]. In our patient’s case, clonazepam, carbamazepine, and phenobarbital were used to suppress such seizures. Those drugs are considered safe for patients with a POLG mutation [9]. Our case supports the recommendation by Saneto et al. [8] to incorporate an early genetic test for POLG mutation in a patient with hepatic encephalopathy from an unknown origin.

Both North American and European guidelines for acute liver failure in pediatrics do not indicate when to test for a mitochondrial genetic disorder in a newborn with hepatic encephalopathy [10,11]. Therefore, it might be recommended that a standardized diagnostic assessment scheme be created.

Treatment for mitochondrial mutations, specifically the POLG mutation, is primarily symptomatic and supportive [12]. Despite many interventions, neither treatment was effective in our patient's case. The continuous lack of response to treatment might suggest a non-reversible etiology of hepatic failure, like a POLG mutation. A prolonged lack of response to best-care treatment might indicate an underlying mitochondrial disease etiology.

Gaudo et al. presented a case of a patient with a Borrelia burgdorferi infection that had exacerbated the underlying POLG mutation [13]. We cannot determine that the positive IgG titer for Borrelia burgdorferi, indicating possible past infection, led to the deterioration of the patient's condition. More research is mandated on infectious diseases provoking POLG-related mitochondrial diseases.

The genetic test on the patient was received post-mortem. The variant of the POLG mutation in this patient, NM_002693.3(POLG):c.3104+2T>A (Variation ID: 422378 Accession: VCV000422378.8), is a single nucleotide mutation located in 15q26.1. Our patient is a male of European (non-Finnish) descent; therefore, the frequency of this variant is one in 350,102 (0.00000286). This variant is likely to be pathogenic, and as far as we know, it is the first time an article about a patient with this variant has been published. The c.3104+2 T>A splice site variant destroys the splice donor site in intron 19. It is expected to cause abnormal gene splicing, leading to an abnormal message, resulting in nonsense-mediated mRNA or an abnormal protein product. This splice site mutation was previously suggested to be related to Alpers-Huttenlocher syndrome (AHS) [14]. The recommended treatment for AHS is symptomatic and palliative. Therefore, the late arrival of the genetic test would not influence the patient's prognosis.

## Conclusions

We want to highlight the complexities associated with diagnosing hepatic encephalopathy in newborns, which demands a thorough diagnostic process. Those complexities are rooted in the clinical nature of the diagnosis and the lack of unique markers to differentiate the possible etiologies. Secondly, we suggest creating a standardized diagnostic scheme for newborns with HE. We recommend a screen test for POLG mutations be included relatively early in the diagnostic process. The genetic test must be performed early in the diagnostic process due to the possibility of administering valproic acid to treat seizures and the potentially hazardous effect of this drug on patients with a POLG mutation. Thirdly, as far as we know, this is the first article about the NM_002693.3(POLG):c.3104+2T>A mutation. Although it was previously documented in genetic databases, clinicians need to know about the possible pathogenicity of such a mutation.

## References

[1] Mas A (2006). Hepatic encephalopathy: from pathophysiology to treatment. Digestion.

[2] Jones EA (2002). Ammonia, the GABA neurotransmitter system, and hepatic encephalopathy. Metab Brain Dis.

[3] Rahman S (2012). Mitochondrial disease and epilepsy. Dev Med Child Neurol.

[4] Saneto RP, Cohen BH, Copeland WC, Naviaux RK (2013). Alpers-Huttenlocher syndrome. Pediatr Neurol.

[5] Shanmugam NP, Bansal S, Greenough A, Verma A, Dhawan A (2011). Neonatal liver failure: aetiologies and management--state of the art. Eur J Pediatr.

[6] Ciocca M, Álvarez F (2017). Neonatal acute liver failure: a diagnosis challenge. Arch Argent Pediatr.

[7] Karadağ N, Okbay Güneş A, Karatekin G (2021). Acute liver failure in newborns. Turk Arch Pediatr.

[8] Saneto RP, Lee IC, Koenig MK, Bao X, Weng SW, Naviaux RK, Wong LJ (2010). POLG DNA testing as an emerging standard of care before instituting valproic acid therapy for pediatric seizure disorders. Seizure.

[9] Lopriore P, Gomes F, Montano V, Siciliano G, Mancuso M (2022). Mitochondrial epilepsy, a challenge for neurologists. Int J Mol Sci.

[10] Squires JE, Alonso EM, Ibrahim SH, Kasper V, Kehar M, Martinez M, Squires RH (2022). North American Society for Pediatric Gastroenterology, Hepatology, and Nutrition position paper on the diagnosis and management of pediatric acute liver failure. J Pediatr Gastroenterol Nutr.

[11] European Association for the Study of the Liver (2017). EASL Clinical Practical Guidelines on the management of acute (fulminant) liver failure. J Hepatol.

[12] Specchio N, Pietrafusa N, Calabrese C (2020). POLG1-related epilepsy: review of diagnostic and therapeutic findings. Brain Sci.

[13] Gaudó P, Emperador S, Garrido-Pérez N (2020). Infectious stress triggers a POLG-related mitochondrial disease. Neurogenetics.

[14] (2024). Varsome. https://varsome.com/variant/hg38/rs747632869.

